# Chemically Attenuated Blood-Stage Plasmodium yoelii Parasites Induce Long-Lived and Strain-Transcending Protection

**DOI:** 10.1128/IAI.00157-16

**Published:** 2016-07-21

**Authors:** Amber I. Raja, Yeping Cai, Jennifer M. Reiman, Penny Groves, Sumana Chakravarty, Virginia McPhun, Denise L. Doolan, Ian Cockburn, Stephen L. Hoffman, Danielle I. Stanisic, Michael F. Good

**Affiliations:** a Institute for Glycomics, Griffith University, Gold Coast, Queensland, Australia; b John Curtin School of Medical Research, Australian National University, Acton, Australian Capital Territory, Australia; c QIMR Berghofer Medical Research Institute, Brisbane, Queensland, Australia; d Sanaria, Inc., Rockville, Maryland, USA; University of South Florida

## Abstract

The development of a vaccine is essential for the elimination of malaria. However, despite many years of effort, a successful vaccine has not been achieved. Most subunit vaccine candidates tested in clinical trials have provided limited efficacy, and thus attenuated whole-parasite vaccines are now receiving close scrutiny. Here, we test chemically attenuated Plasmodium yoelii 17X and demonstrate significant protection following homologous and heterologous blood-stage challenge. Protection against blood-stage infection persisted for at least 9 months. Activation of both CD4^+^ and CD8^+^ T cells was shown after vaccination; however, *in vivo* studies demonstrated a pivotal role for both CD4^+^ T cells and B cells since the absence of either cell type led to loss of vaccine-induced protection. In spite of significant activation of circulating CD8^+^ T cells, liver-stage immunity was not evident. Neither did vaccine-induced CD8^+^ T cells contribute to blood-stage protection; rather, these cells contributed to pathogenesis, since all vaccinated mice depleted of both CD4^+^ and CD8^+^ T cells survived a challenge infection. This study provides critical insight into whole-parasite vaccine-induced immunity and strong support for testing whole-parasite vaccines in humans.

## INTRODUCTION

Malaria is a major global health burden, with nearly half of the human population at risk of becoming infected with Plasmodium parasites (1). In 2015 there were an estimated 214 million clinical cases and a malaria-associated death toll of approximately 438,000 (1). Although malaria control measures, such as insecticide-treated nets, indoor residual spraying, and antimalaria drug treatment, have contributed to a reduction in morbidity and mortality (1), additional strategies, such as a vaccine, will be required for the continued reduction and eventual eradication of human malaria.

The development of malaria vaccines has largely focused on a limited number of recombinant subunit vaccine candidates, immune responses to which have shown an association with protection in immunoepidemiological studies (2–4). Many of these immune targets, such as merozoite surface proteins 1 and 2 that are involved in the invasion of red blood cells (RBCs), are encoded by multiple alleles. The genetic diversity of Plasmodium, especially allelic polymorphism, has been a major stumbling block for subunit vaccine candidates, and this has undoubtedly impacted their ability to provide sustained protection in endemic settings (5–7). The most advanced vaccine candidate, RTS,S, targets the preerythrocytic stage of the parasite's life cycle. This subunit vaccine candidate is based on the Plasmodium falciparum circumsporozoite surface protein. Data from clinical trials suggest that RTS,S is only able to induce temporary immunity and is unable to provide protection to residents of regions of endemicity over a sustained period of time (8–12). Therefore, efforts in malaria vaccine development need to continue in order to develop more efficacious vaccines.

An alternative approach to malaria vaccine development is to use the whole parasite. This is now being vigorously pursued for the sporozoite stage of the life cycle with significant initial success (13, 14). A vaccine approach using whole blood-stage parasites is advantageous as it includes a broad array of antigens, including those that are conserved between different parasite strains and species and, if successful, will reduce the morbidity and mortality of malaria, even if does not completely prevent infection. The inclusion of conserved antigens may result in the induction of a protective strain- and species-transcending immune response and therefore result in improved efficacy compared with subunit vaccine candidates. Protection induced by whole blood-stage malaria parasites has been studied using infection and drug-cure systems (15–17); these data provide a strong rationale for the development of a whole-parasite vaccine. Attenuation of the malaria parasite, such as by chemical treatment using DNA-binding drugs, has provided the means for investigating this approach (18–20). In a rodent model, vaccination with chemically attenuated synchronous Plasmodium chabaudi ring-stage parasites was shown to protect mice against parasite challenge (20). Although these results are encouraging, they do not indicate that attenuated whole blood-stage parasites will provide a general strategy for vaccine development, in particular for parasites where cellular tropism and sequestration differ and where different mechanisms of immunity are known to be relevant. Plasmodium yoelii, like the human parasite Plasmodium vivax, has a tropism for reticulocytes. Mature forms of both parasites are also found in the peripheral blood, unlike P. falciparum and P. chabaudi, where only immature ring forms are found in peripheral blood. In addition, the mechanism of immunity for P. yoelii differs from that for P. chabaudi. Immunity to P. chabaudi after infection and drug-cure is thought to be mediated predominantly by T cells (21), although antibodies are known to play a role later in protection (22). Conversely, antibodies are thought to play a primary role in immunity to P. yoelii (23). Furthermore, in some parasite species, the activation of cellular immune responses was shown to be associated with the induction of pathology (24). These various observations indicate the need for further preclinical investigations of the attenuated blood-stage malaria vaccine strategy.

Here, we have used the DNA-binding drug centanamycin to attenuate blood-stage P. yoelii 17X parasites and investigated the protective efficacy of this vaccine. Since P. yoelii exists asynchronously during the blood-stage of its life cycle (25), we were able to investigate a blood-stage vaccine expressing antigens from all stages of asexual blood-stage parasite development. We found that the attenuated vaccine induced profound stage-specific immunity and long-lived immunological memory, with protection dependent on both CD4^+^ T cells and B cells. Cell depletion studies revealed that CD8^+^ T cells could play a significant role in pathogenesis.

## MATERIALS AND METHODS

### Ethics statement.

Animal work was approved by the Griffith University Animal Ethics Committee under approvals BDD/07/10 and GLY/05/12. Sporozoite challenge by mosquito bite and intravenous injection of freshly dissected sporozoites was approved by the Australian National University Animal Experimentation Ethics Committee under approval A2013/12. All animal care and use protocols adhered to the Australian Code of Practice for the Care and Use of Animals for Scientific Purposes.

### Mice.

Six- to eight-week-old female BALB/c, C57BL/6, and μMT mice were used. BALB/c and C57BL/6 mice were obtained from Animal Resource Centre, Perth, Australia. μMT mice were originally obtained from the Jackson Laboratory (Bar Harbor, ME) and were backcrossed on the C57BL/6 background for >10 generations. Mice were housed under PC2 and QAP (where applicable) regulations.

### Malaria parasites.

The nonlethal phenotype of P. yoelii 17XNL was not always stable between experiments; therefore, rather than refer to the parasite as P. yoelii 17XNL or 17XL, here, the strain is referred to as P. yoelii 17X. Blood-stage P. yoelii 17X and P. yoelii YM strains were obtained from Richard Carter (Edinburgh, United Kingdom) and maintained by serial passage in inbred and outbred mice. P. yoelii 17X sporozoites that were used in the mosquito bite challenge and freshly dissected sporozoite challenge studies were obtained from Fidel Zavala (Johns Hopkins University, Baltimore, MD) under Australian quarantine guidelines. Cryopreserved P. yoelii 17X sporozoites (26, 27) were obtained from Stephen Hoffman (Sanaria, Inc., Rockville, MD) under Australian quarantine guidelines.

### Attenuation and vaccination of mice.

For parasite attenuation, P. yoelii 17X-infected blood was collected into EDTA or lithium-heparin blood collection tubes (BD Biosciences). Blood was diluted to 10% (vol/vol) in serum-free RPMI 1640 (Gibco). The attenuating drug, centanamycin, was dissolved in dimethyl sulfoxide (Sigma-Aldrich), PET (6 parts of polyethylene glycol 400 [Sigma-Aldrich], 3 parts of 100% ethanol [Sigma-Aldrich], and 1 part of Tween 80 [Merck]) and 5% glucose (Sigma-Aldrich) to produce a 2 mM stock. A final concentration of 2 μM centanamycin was used to attenuate parasites. Parasites were incubated with the attenuating drug for 40 min (with agitation every 10 min) at 37°C in CO_2_. Treated parasitized RBCs (pRBCs) were washed twice with RPMI 1640 and once with phosphate-buffered saline (PBS) at 277 × *g* for 10 min. The cells were resuspended in 0.9% saline (Pfizer) or PBS. Viable cells were counted based on the exclusion of dead cells by trypan blue (0.1% [wt/vol] in PBS). The immunizing dose of 10^6^ pRBCs was calculated, and mice were injected intravenously. Control groups received 10^6^ normal RBCs (nRBCs) that were treated with centanamycin as described above.

### Challenge of mice with blood-stage parasites or sporozoites and monitoring of disease.

For blood-stage parasite challenge, control and/or vaccinated mice were challenged by intravenous injection with 10^5^ P. yoelii 17X or P. yoelii YM pRBCs in 0.9% saline (Pfizer) or PBS.

For sporozoite challenge by mosquito bite, mice were anesthetized using ketamine (100 mg/kg)-xylazine (10 mg/kg) and laid over a cage containing 50 P. yoelii 17X-infected mosquitoes. Each mouse received ∼10 mosquito bites over 30 min. Livers from mice were removed 40 to 42 h after sporozoite challenge.

For challenge with freshly dissected sporozoites by intravenous injection, P. yoelii 17X sporozoites were dissected from the salivary glands of infected mosquitoes at between 14 and 21 days postinfection. The number of sporozoites was determined using a counting chamber, and the sporozoites were diluted in PBS containing 1% heat-inactivated naive mouse serum. Mice were injected intravenously with 2,000 sporozoites in a volume of 200 μl. Livers from mice were removed 40 to 42 h after sporozoite injection.

For challenge by intravenous injection of cryopreserved sporozoites, P. yoelii 17X sporozoites (Sanaria, Inc.) were thawed in a 30°C water bath for 30 s. The vial was centrifuged briefly, and sporozoites were diluted in PBS containing 5% naive mouse serum. Mice were injected intravenously with 4,000 sporozoites in a volume of 200 μl. Infection in mice was either followed through to blood-stage infection or the livers were removed from mice 40 to 42 h after sporozoite injection.

After challenge, the mice were monitored every other day by examining stained thin blood films and every 4 days by measuring hemoglobin levels (HemoCue 201^+^ Analyser; Hemocue). To reduce observer bias, all experiments, except for the T cell depletion study, were monitored with the observer blind to the identification of the experimental groups. Clinical scoring criteria (described in Table S1 in the supplemental material) were used to monitor disease every 2 days. Mice that showed signs of severe disease were euthanized using CO_2_ gas or by cervical dislocation.

### Detection of IgG against crude parasite antigen by enzyme-linked immunosorbent assay.

Blood was collected from mice at specific time points pre- and postchallenge and diluted 1 in 20 in PBS. The samples were centrifuged at 8,928 × *g* for 5 min, and the supernatant was removed and stored at −80°C. Ninety-six-well MaxiSorp Nunc immunoplates (Nunc) were coated with 10 μg/ml of crude parasite antigen in coating buffer for 2 h at room temperature. Plates were blocked with 5% skim milk in PBS overnight at 4°C. Serum samples were used at a 1 in 20 dilution or titrated using 3-fold dilutions from 1 in 50 to 1 in 109,350. Samples were dispensed at a volume of 50 μl per well and incubated for 2 h at room temperature. For the assessment of memory B cell responses, a dilution of 1 in 1,350 of the titrated sera was chosen as a representation of the data. After five washes with PBS–0.05% Tween 20 (Merck), the plates were incubated with goat anti-mouse IgG/horseradish peroxidase-conjugated antibody (1:3,000 in blocking buffer) (Bio-Rad) for 2 h. Plates were washed a further five times and then incubated with tetramethylbenzidine substrate (BD Biosciences) for 15 min. The reaction was stopped using 1 M sulfuric acid, and the plates were read using a Victor 3 plate reader (Perkin-Elmer) at 450 nM.

### Analysis of vaccine-induced CD4^+^ T follicular helper cells.

Spleens were removed from mice, broken down manually, and then lysed with Geys lysis buffer. The splenocytes were washed with MACS buffer (PBS supplemented with bovine serum albumin and EDTA) at 277 × *g* for 5 min and dispensed at 3 × 10^6^ cells per well in MACS buffer into V-bottom 96-well plates (Sarstedt). The cells were incubated in 100 μl of Fc receptor block (from cell line 2.4G2; ATCC) for 10 min on ice and centrifuged at 177 × *g* for 3 min. The supernatants were removed, and the cells were resuspended and incubated with CD4-fluorescein isothiocyanate (CD4-FITC) (clone GK1.5; BD Pharmingen), CD3-V450 (clone 17A2; BD Pharmingen), and CXCR5-biotin (clone 2G8; BD Pharmingen) on ice for 20 min. The cells were washed twice in MACS buffer. Streptavidin-allophycocyanin (streptavidin-APC) (BD Pharmingen) was added to each well, and the cells were incubated for 15 min on ice and washed at 177 × *g* for 3 min. Fix/Perm buffer (eBioscience) was added, and the cells were incubated for 30 min on ice. The cells were washed twice with Perm/Wash buffer (eBioscience) at 177 × *g* for 3 min, the supernatants were removed, and the cells were resuspended. Bcl6-PE (clone K112-91; BD Pharmingen) was added to each well, and the cells were incubated on ice for an hour. The cells were washed and resuspended in Perm/Wash buffer for analysis on an LSR Fortessa flow cytometer (BD Biosciences). Data analysis was performed using FACSDiva software version 6 (BD Biosciences) and FlowJo software version 7.6.5 (FlowJo, LLC). The gating strategy in Fig. S1 in the supplemental material was used to identify T follicular helper (Tfh) cells.

### Assessment of T cell activation.

Fifty microliters of blood was collected from vaccinated and control mice on day 7 after each injection. Blood was collected into 1 ml of 5 mM EDTA (Gibco) in PBS. Samples were centrifuged at 400 × *g* for 5 min, and the supernatants were removed. One milliliter of ammonium-chloride-potassium (ACK) lysis buffer was added per sample, followed by incubation for 5 min at room temperature. The samples were centrifuged at 400 × *g* for 5 min and supernatants were removed. Samples were resuspended in 100 μl of MACs buffer and transferred to a V-bottom 96-well plate (Sarstedt). The samples were centrifuged and resuspended in 60 μl of Fc receptor block (from cell line 2.4G2; American Type Culture Collection [ATCC]) and incubated for 10 min on ice. The samples were centrifuged, and 50 μl of an antibody mastermix containing CD4 (RM4-5, V500; BD Horizon), CD8 (53.6.7, peridinin chlorophyll protein [PerCP]-Cy5.5; BD Pharmingen), CD11a (2D7, FITC, BD Pharmingen), and CD49d (R1-2, phycoerythrin [PE]; BD Pharmingen) in MACS buffer was added per well. The cells were incubated on ice for 20 min and washed three times in MACS buffer by centrifugation at 400 × *g* for 5 min. The cells were then resuspended in 300 μl of MACS buffer. Samples were analyzed using the LSR Fortessa flow cytometer (BD Biosciences), FACSDiva software version 6 (BD Biosciences), and FlowJo software version 7.6.5 (FlowJo, LLC). The gating strategy in Fig. S2 in the supplemental material was used to identify activated T cells.

### Depletion of CD4^+^ and CD8^+^ T cells.

Following a regimen of three vaccine doses, mice were depleted of CD4^+^ T cells, CD8^+^ T cells, or both CD4^+^ and CD8^+^ T cells. Depletions were undertaken using intraperitoneal injections of anti-CD4 antibodies from the supernatant of the clone GK1.5 (Bio-x-cell) at 0.25 mg per mouse (depletion of CD4^+^ T cells), anti-CD8 antibodies from supernatant of the clone 53.5.8 (Bio-x-cell) at 0.5 mg per mouse (depletion of CD8^+^ T cells), or a combination of 0.25 mg per mouse of anti-CD4 and 0.5 mg per mouse of anti-CD8 antibodies (depletion of CD4^+^ and CD8^+^ T cells) from day −3 to day 0 relative to challenge on day 0. The gating strategy in Fig. S3 in the supplemental material was used to identify CD4 and CD8 T cells. Depletions were maintained by continued injections of the relevant antibodies on day 7, 15, and 22 relative to challenge on day 0. A control group of vaccinated mice received intraperitoneal injections of 0.5 mg per mouse rat Ig antibodies (Sigma-Aldrich) at each time point.

### Splenocyte proliferation and cytokine assays.

Spleens were removed from mice, broken down manually, and then lysed with Geys lysis buffer. Splenocytes were washed with complete RPMI medium (RPMI supplemented with 10% heat-inactivated fetal bovine serum, 1% l-glutamine [100×], 1% penicillin-streptomycin, and 0.1% 2-mercaptoethanol) at 277 × *g* for 5 min and dispensed at 4 × 10^5^ cells per well in complete medium into U-bottom 96-well plates (BD Falcon). Cultures were grown for 72 h at 37°C and 5% CO_2_ in the presence of complete medium (negative control), 5 × 10^6^ normal RBCs from a naive mouse (nRBCs)/ml (negative control), 10 μg of concanavalin A (Sigma-Aldrich)/ml (a T cell-specific mitogen, positive control), or 5 × 10^6^ pRBCs/ml.

To measure the cytokines secreted by cultured splenocytes, culture supernatants were removed after 54 h, aliquoted, and stored at −80°C just prior to the addition of [^3^H]thymidine. Interleukin-2 (IL-2), IL-4, IL-6, IL-10, IL-17α, gamma interferon (IFN-γ), and tumor necrosis factor (TNF) were measured in culture supernatants using a Th1/Th2/Th17 CBA kit (BD Biosciences) according to the manufacturer's instructions with the following modifications. One hundred eighty microliters of assay diluent was added to a 20-μl aliquot of reconstituted standard. Serial dilutions of the standard were made. Then, 20 μl of a master mix containing 2 μl of each vortexed capture bead per sample and an equal volume of PE detection reagent was added per well to a V-bottom 96-well plate (Sarstedt). Next, 10 μl of the standards and samples was added to the appropriate wells and incubated for 2 h at room temperature. The wells were washed with 150 μl of wash buffer and centrifuged at 177 × *g* for 3 min. The supernatants were removed, and the samples were resuspended in 200 μl of wash buffer. The samples were analyzed using a LSR Fortessa flow cytometer (BD Biosciences), FACSDiva software version 6 (BD Biosciences), and FCAP Array software version 1.0.1 (BD Biosciences).

The uptake of [^3^H]thymidine (radioisotope-labeled nucleoside; Perkin-Elmer) was used to assess splenocyte proliferation. Cultured spleen cells were pulsed with 1 μCi of [^3^H]thymidine per well for the last 18 h of the 72-h culture period. Plates were stored at −80°C prior to thawing and harvesting of cells onto glass fiber filter mats (Perkin-Elmer). Radioisotope incorporation was estimated by β-emission spectroscopy using a MicroBeta^2^ β counter (Perkin-Elmer).

### Quantification of liver-stage parasites by RT-qPCR. Method 1.

For method 1, the livers were removed from mice 40 to 42 h after mosquito bite or intravenous injection with freshly dissected sporozoites. RNA was extracted from the liver as previously described (28). cDNA synthesis was carried out using an iScript cDNA synthesis kit (Bio-Rad) in accordance with the manufacturer's instructions. Reverse transcription-quantitative PCR (RT-qPCR) to determine the level of P. yoelii 18S rRNA was performed using the forward primer NYU-Py3 (5′-GGGGATTGGTTTTGACGTTTTTGCG-3′) and the reverse primer NYU-Py5 (5′-AAGCATTAAATAAAGCGAATACATCCTTAT-3′) (29). Briefly, the Py18S PCR was conducted using Power SYBR Green PCR master mix (Applied Biosystems), 0.5 μM concentrations (each) of forward and reverse primer, and 1 μl of template (negative controls, unknown samples, positive control, or standard curve). The PCR was performed using a thermal profile of 50°C for 2 min, followed by 95°C for 10 min, followed by 40 cycles of 95°C for 15 s and 60°C for 1 min, and then terminated by 95°C for 15 s, 60°C for 1 min, 95°C for 15 s, and 60°C for 15 s on an Applied Biosystems 7500 real-time PCR system. A PCR for the housekeeping gene GAPDH (glyceraldehyde-3-phosphate dehydrogenase) using the forward primer (5′-GTTGTCTCCTGCGACTTCA) and reverse primer (5′-GGTGGTCCAGGGTTTCTTA) was run to control for sample to sample variation; cycling conditions and other parameters were the same as for the P. yoelii 18s rRNA reaction.

### Method 2.

For method 2, the livers were removed from mice 40 to 42 h after intravenous injection of cryopreserved sporozoites. RNA was extracted from the livers as previously described (30). The concentration and purity of RNA were measured using a Nanodrop2000c spectrophotometer (Thermo Scientific). cDNA synthesis was carried out using a SuperScript VILO cDNA synthesis kit (Invitrogen) in accordance with the manufacturer's instructions. RT-qPCR to determine the level of P. yoelii 18S RNA was performed using the forward primer Py685F (5′-CTTGGCTCCGCCTCGATAT), the reverse primer Py782R (5′-TCAAAGTAACGAGAGCCCAATG), and a probe (6-carboxyfluorescein [FAM]-CTGGCCCTTTGAGAGCCCACTGATT-BHQ-1) (31). Briefly, the Py18S PCR was conducted using TaqMan Fast Advanced Master mix (Applied Biosystems), 1 μM concentrations (each) of forward and reverse primers, 0.25 μM probe (Sigma-Aldrich), and 2 μl of template (negative controls, unknown samples, positive control, or standard curve). The PCR was run using a thermal profile of 50°C for 2 min and 95°C for 2 min, followed by 50 cycles of 95°C for 5 s and 60°C for 30 s on the FAM channel using a Bio-Rad (CFX96) real-time PCR machine. A PCR for the GAPDH housekeeping gene was run to control for sample-to-sample variation. Briefly, the PCR was conducted using a GAPDH kit (Mm03302249_g1, Applied Biosystems), Platinum *Taq* (Life Technologies), 200 μM deoxynucleoside triphosphates (Promega), and 2 μl of template (negative controls, unknown samples, positive control, or standard curve). The PCR was run using a thermal profile of 95°C for 2 min, followed by 45 cycles of 95°C for 5 s and 60°C for 30 s on the FAM channel using the Bio-Rad (CFX96) real-time PCR machine.

### Statistical analysis.

All statistical analyses were conducted using GraphPad Prism software version 6. An unpaired, two-tailed *t* test was used when comparing two experimental groups. For experiments comparing more than two groups, a one-way analysis of variance (ANOVA) was used, followed by Tukey's multiple-comparison tests.

## RESULTS

### Vaccine-induced immune protection against homologous and heterologous parasites.

**FIG 1:**
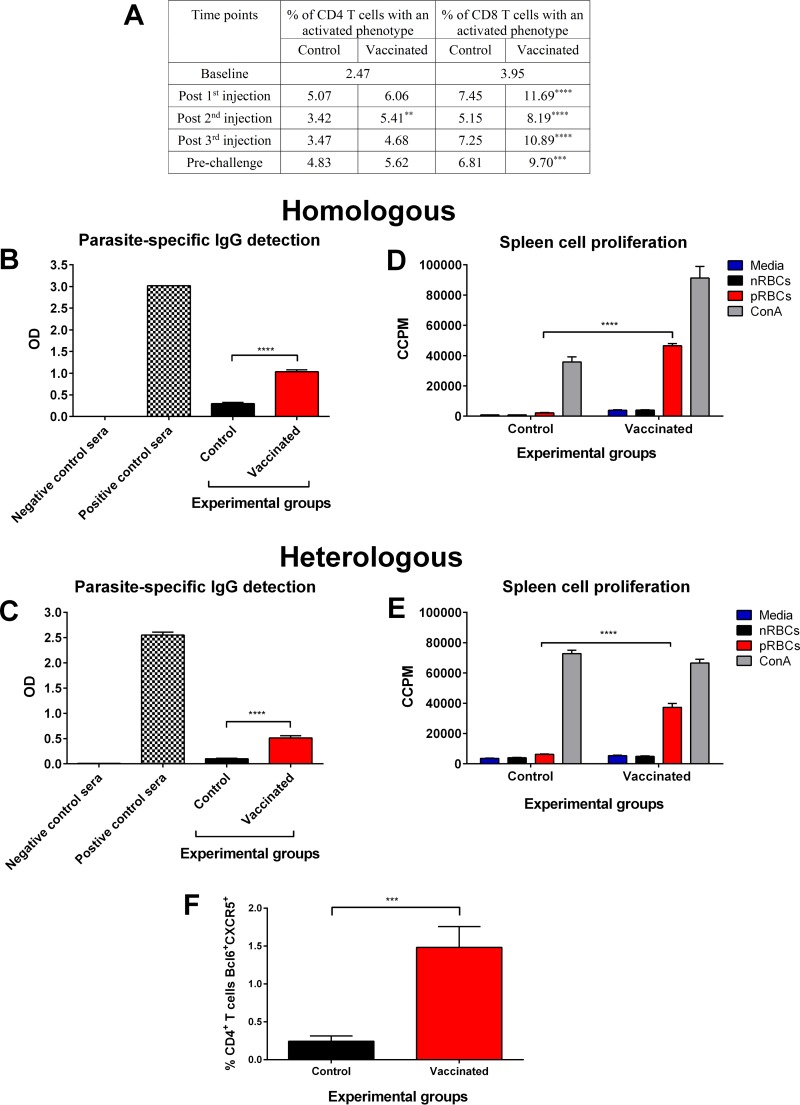
Induction of immune responses after vaccination with chemically attenuated P. yoelii 17X pRBCs. (A) Vaccine-induced activation of circulating CD4^+^ and CD8^+^ T cells. CD49d^hi^ CD11a^hi^ markers were used to identify activated CD4^+^ T cells, and CD8^lo^ CD11a^hi^ markers were used to identify activated CD8^+^ T cells in the blood of vaccinated and control mice 7 days after each injection. Data represent the mean results from 20 BALB/c mice per group. Baseline data represent mice from both groups prior to injection. (B and C) Induction of IgG to P. yoelii 17X (B) or P. yoelii YM (C) antigens after three doses of 10^6^ chemically treated P. yoelii 17X pRBCs or nRBCs. IgG was measured immediately prior to challenge using sera diluted 1 in 20, and each sample was tested in duplicate. Sera from naive mice and P. yoelii 17X or P. yoelii YM infection-drug-cured mice were included as negative and positive controls, respectively. Results are represented as the optical density (OD) at 450 nm. Each group contained 10 BALB/c mice, and error bars represent the standard errors of the mean (SEM) for each group. (D and E) Induction of splenocyte proliferation to fresh P. yoelii 17X pRBCs (D) or P. yoelii YM pRBCs (E) after three doses of 10^6^ chemically treated P. yoelii 17X pRBCs or nRBCs. Proliferation was evaluated by incorporation of [^3^H]thymidine and measured as corrected counts per minute (CCPM). Each group contained three BALB/c mice, and error bars represent the SEM for each group. (F) Induction of CD4^+^ Tfh cells after vaccination. The percentage of CD4^+^ T cells expressing Tfh markers (Bcl6 and CXCR5) was assessed in the spleens of vaccinated and control mice 2 days after the third dose. Each group contained 10 BALB/c mice, and error bars represent the SEM for each group. **, *P* = 0.0028; ***, *P* < 0.001; ****, *P* < 0.0001. An unpaired, two-tailed *t* test was used to compare two experimental groups. For experiments comparing more than two groups, a one-way ANOVA was used, followed by Tukey's multiple-comparison tests.

A regimen of three vaccine doses of 10^6^ chemically attenuated P. yoelii 17X-parasitized RBCs (pRBCs) was used to vaccinate BALB/c mice. The control group received chemically treated normal RBCs (nRBCs) at each time point. We sought to determine whether vaccination induced activation of CD4^+^ and CD8^+^ T cells using CD49d and CD11a as markers of recent activation (32, 33). Overall, circulating CD4^+^ T cells showed limited activation in vaccinated mice compared to control mice (Fig. 1A). Significant activation of CD8^+^ T cells was identified in the blood of mice vaccinated with chemically attenuated blood-stage P. yoelii 17X parasites compared to control mice (*P* ≤ 0.0001).

Prior to challenge, there was significantly higher P. yoelii 17X-specific IgG in the sera of vaccinated mice than in the sera of control mice (*P* < 0.0001) (Fig. 1B). We observed that these antibodies cross-reacted with the heterologous strain, P. yoelii YM (Fig. 1C).

Immediately prior to challenge, a subset of mice from each group was sacrificed to assess splenocyte proliferation in response to homologous parasites. Parasite-specific cellular proliferation was significantly higher for spleen cells from vaccinated mice than for spleen cells from control mice (Fig. 1D) (*P* < 0.0001). The proliferative response from vaccinated mice was also significantly higher toward the heterologous parasite (*P* < 0.0001) (Fig. 1E).

Follicular helper cells are a distinct subset of CD4^+^ T cells that are important in the stimulation of B cells in germinal centers and in the induction of long-lived antibody responses (34). Consistent with the immunoglobulin response of vaccinated mice, we observed a higher percentage of CD4^+^ T follicular helper (Tfh) cells (Bcl6^+^ CXCR5^+^) in the spleens of vaccinated mice (1.48% ± 0.27%) than in spleens of control mice (0.24% ± 0.07%; *P* = 0.0006) (Fig. 1F). These data thus demonstrate the induction of both cellular and humoral immune responses after vaccination.

**FIG 2:**
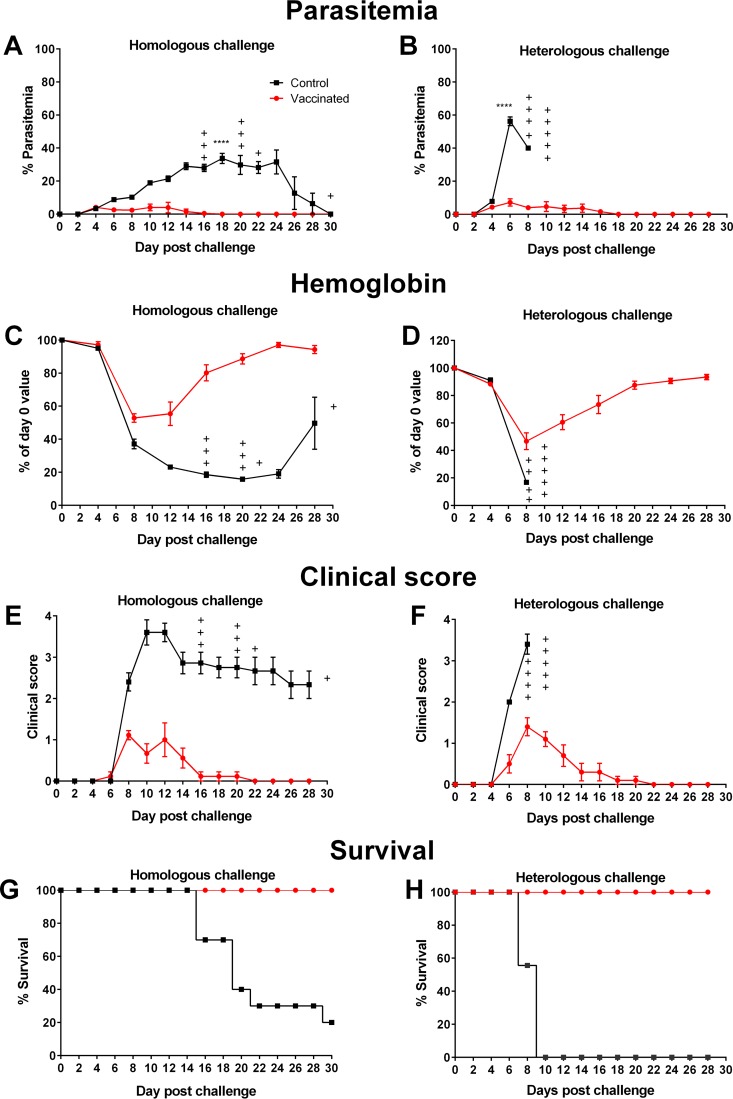
Vaccine-induced protection against homologous and heterologous strain challenge. After three doses of 10^6^ chemically treated P. yoelii 17X pRBCs or nRBCs, BALB/c mice were challenged with 10^5^ P. yoelii 17X pRBCs (left panels) or P. yoelii YM pRBCs (right panels). After challenge, parasitemias (A and B), hemoglobin levels (C and D), clinical scores (E and F), and survival (G and H) were assessed. The vaccinated group challenged with P. yoelii 17X contained nine mice; all other groups contained ten mice. Error bars represent the SEM for each group. +, mice that succumbed to infection. Peak parasitemia was significantly lower in vaccinated mice than in control mice when challenged with homologous or heterologous parasites (****, *P* < 0.0001). Data were analyzed using an unpaired, two-tailed *t* test.

At 4 weeks after vaccination, mice were challenged with 10^5^ P. yoelii 17X pRBCs or P. yoelii YM pRBCs. Mice were strongly protected against challenge with both strains. After P. yoelii 17X challenge, peak parasitemia for control mice (33.71% ± 3.12%) was reached on day 18 (in the surviving mice) (Fig. 2A). The peak parasitemia for vaccinated mice (4.11% ± 0.40%) was reached on day 4 and was significantly lower than for control mice (*P* < 0.0001). The peak parasitemia of control and vaccinated mice challenged with heterologous parasites was reached on day 6 postchallenge. However, vaccinated mice challenged with P. yoelii YM had a significantly lower peak parasitemia (7.18% ± 2.22%) compared to mice in the control group (56.26% ± 2.67%; *P* < 0.0001) (Fig. 2B). Reduced disease severity was observed in vaccinated mice compared to control mice after challenge with homologous and heterologous parasites, as measured by hemoglobin values, clinical scores, and survival (Fig. 2C to H).

**FIG 3:**
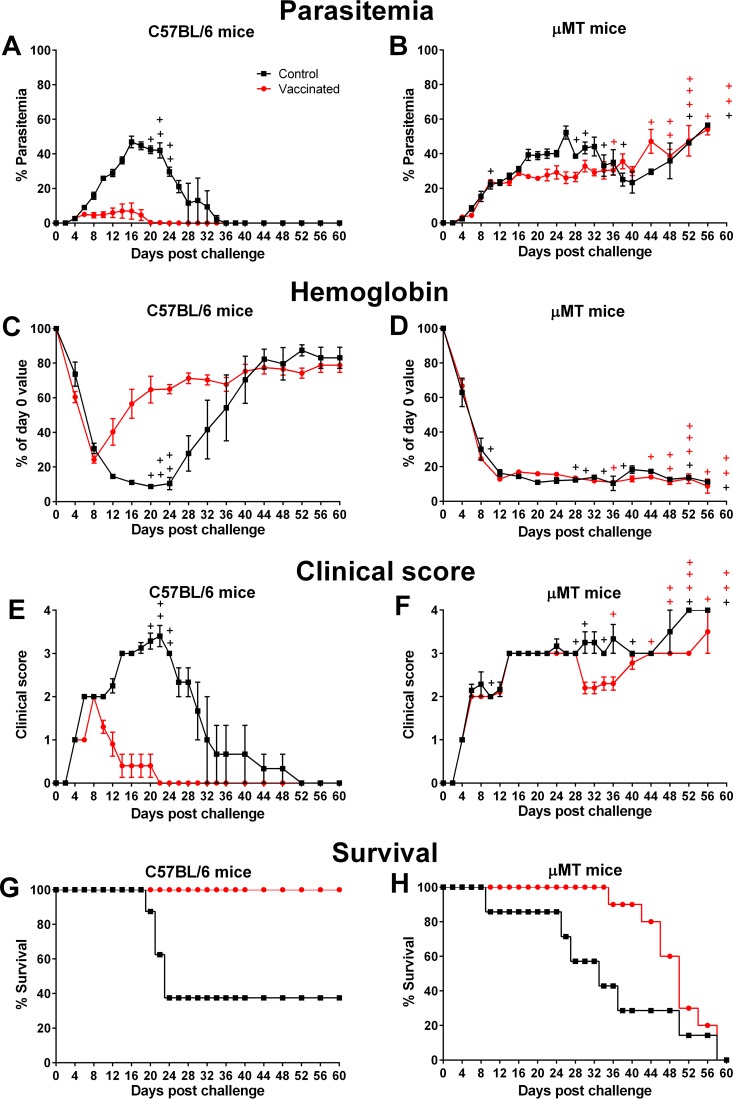
Assessing the role of B cells in vaccine-induced protection following homologous challenge. Following 3 doses of 1 × 10^6^ chemically treated P. yoelii 17X pRBCs or nRBCs, C57BL/6 mice (left panels) and μMT mice (right panels) were challenged with 1 × 10^5^ P. yoelii 17X pRBCs. After challenge, parasitemias (A and B), hemoglobin levels (C and D), clinical scores (E and F), and survival (G and H) were assessed. The control μMT group contained seven mice, the control C57BL/6 group contained eight mice, and both vaccinated groups contained ten mice. Error bars show the SEM for each group. +, mice that succumbed to infection.

To investigate the role of B cells in protection, the responses of immunocompromised μMT mice were studied. B cell development is blocked in the bone marrow of these mice, resulting in a complete deficiency of mature B cells and a lack of antibodies (35). μMT mice were vaccinated with chemically attenuated P. yoelii 17X pRBCs. A control group of μMT mice received chemically treated nRBCs at each vaccination. Vaccinated immunocompetent C57BL/6 mice were also included. Sera from vaccinated immunocompetent C57BL/6 mice collected immediately prior to challenge contained parasite-specific IgG, whereas sera from vaccinated μMT mice did not (data not shown). Mice were challenged 4 weeks after the final vaccination. After challenge, vaccinated immunocompetent mice were protected with a mean peak parasitemia of 1.62% ± 0.33% and reduced disease severity (Fig. 3A, C, E, and G). Vaccinated μMT mice all succumbed to infection by day 60, similarly to unvaccinated μMT mice. Vaccinated mice lived up to 20 days longer than unvaccinated mice, despite a similar course of parasitemia (Fig. 3B and H). The disease profiles were similar for vaccinated and control μMT mice (Fig. 3D and F).

**FIG 4:**
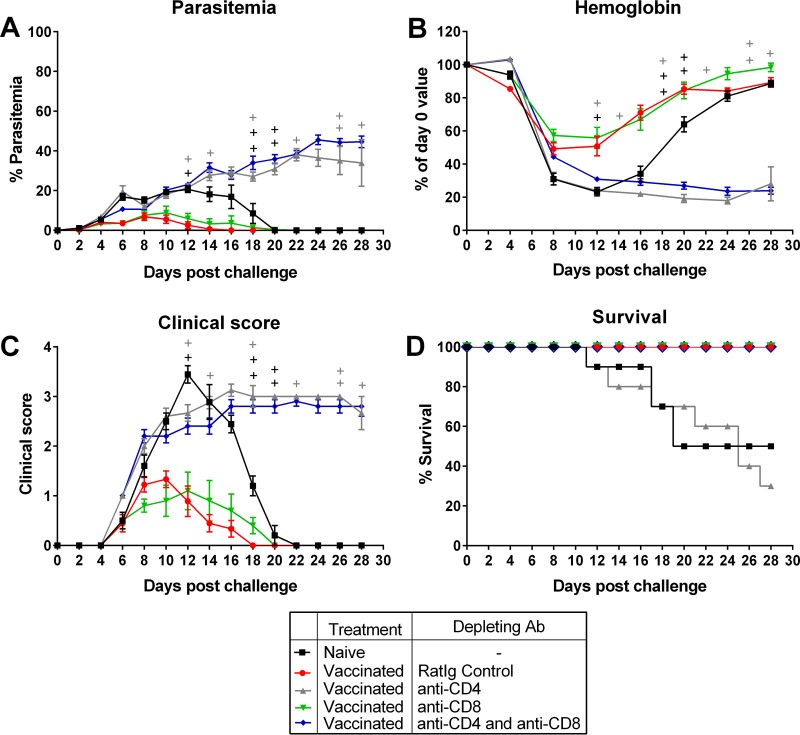
Assessing the role of CD4^+^ and CD8^+^ T cells in vaccine-induced protection following homologous challenge. (A to D) Parasitemia (A), hemoglobin levels (B), clinical scores (C), and survival (D) in BALB/c mice that received three doses of 10^6^ chemically attenuated P. yoelii 17X pRBCs, followed by intraperitoneal injections of rat Ig, anti-CD4, anti-CD8, or a combination of anti-CD4 and anti-CD8 antibodies on days −3, −2, −1, 0, 7, 15, and 22 relative to challenge with 10^5^ P. yoelii 17X pRBCs on day 0. A naive control group was also included. The group that received rat Ig contained nine mice; all other groups contained ten mice. Error bars show the SEM for each group. +, mice that succumbed to infection.

To determine the role of CD4^+^ and CD8^+^ T cells in vaccine-induced protection, vaccinated BALB/c mice were given intraperitoneal injections of rat immunoglobulin (control), anti-CD4, anti-CD8, or a combination of anti-CD4 and anti-CD8 antibodies. At the time of challenge, >95% depletion of the T cell subsets was achieved (data not shown). We observed that depletion of CD4^+^ T cells alone, or depletion of both CD4^+^ and CD8^+^ T cells, rendered the mice unable to control parasitemia (Fig. 4A). These mice also had very poor clinical scores and became anemic (Fig. 4B and C). However, all mice depleted of both cell types survived for the duration of the study (28 days), unlike mice depleted of just CD4^+^ T cells (Fig. 4D). Vaccinated mice depleted of CD8^+^ T cells alone were able to control parasite growth as effectively as vaccinated mice that received control rat Ig and survived for the duration of the experiment (Fig. 4A and D). The data thus show that while CD4^+^ T cells control parasite density, CD8^+^ T cells not only do not control parasite growth but contribute to a poor outcome in the absence of CD4^+^ T cells.

### Sustained immunological memory.

**FIG 5:**
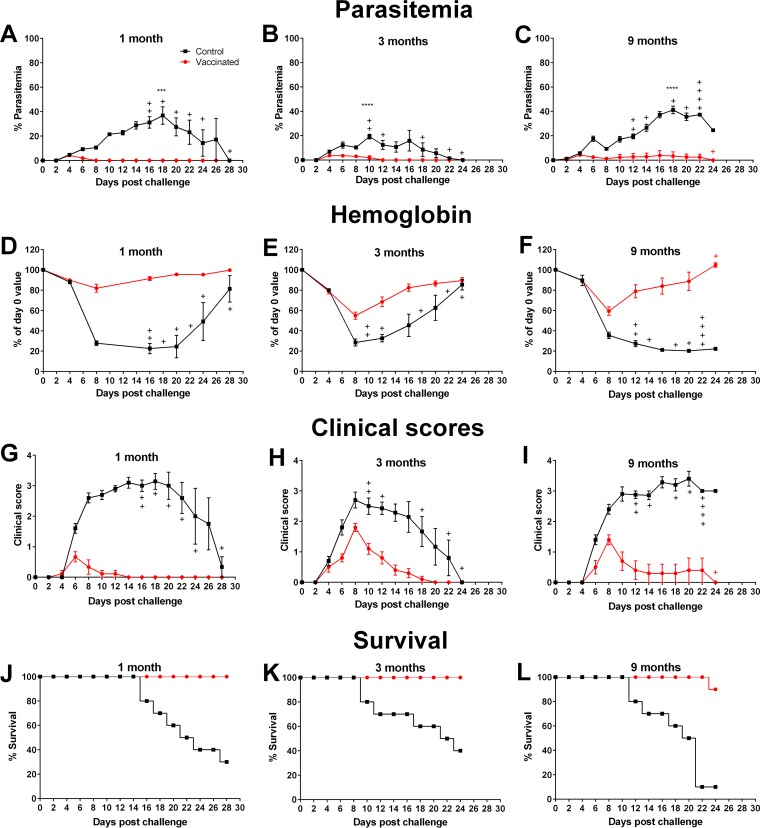
Assessing the duration of vaccine-induced protection after homologous challenge. (A to C) Parasitemia levels in BALB/c mice that received three doses of 10^6^ chemically treated nRBCs (control) or P. yoelii 17X pRBCs (vaccinated) prior to challenge with 10^5^ P. yoelii 17X pRBCs at 1 month (A), 3 months (B), or 9 months (C) after the final dose. Disease severity was evaluated in control and vaccinated mice according to hemoglobin levels (D to F), clinical scores (G to I), and survival (J to L) after homologous challenge at 1 month (left panels), 3 months (middle panels), or 9 months (right panels) after the final dose. The groups challenged at 1 month contained nine mice; all other groups contained ten mice. Error bars show the SEM for each group. +, mice that succumbed to infection. Peak parasitemia was significantly lower in vaccinated mice than in control mice at each time point (***, *P* = 0.0001; ****, *P* < 0.0001). Data were analyzed using an unpaired, two-tailed *t* test.

To assess the duration of protection, vaccinated BALB/c mice were challenged at 3 or 9 months after the final vaccination. Age-matched control mice that received chemically treated nRBCs were also challenged at each time point. Peak parasitemias among vaccinated mice over the course of infection were significantly lower than among their control counterparts at each time point (*P* ≤ 0.0001) (Fig. 5A to C). Vaccinated mice challenged at 3 and 9 months after their final vaccination showed mean parasite densities that were comparable to those seen with mice challenged at 1 month. Also, the disease and survival parameters were better in the vaccinated cohorts than in their control groups at all challenge time points (Fig. 5D to L). Immediately prior to challenge at each time point, there was no difference in memory CD4^+^ or CD8^+^ T cells (CD44^hi^ CD62L^+^) in the blood of vaccinated and control mice (data not shown).

**FIG 6:**
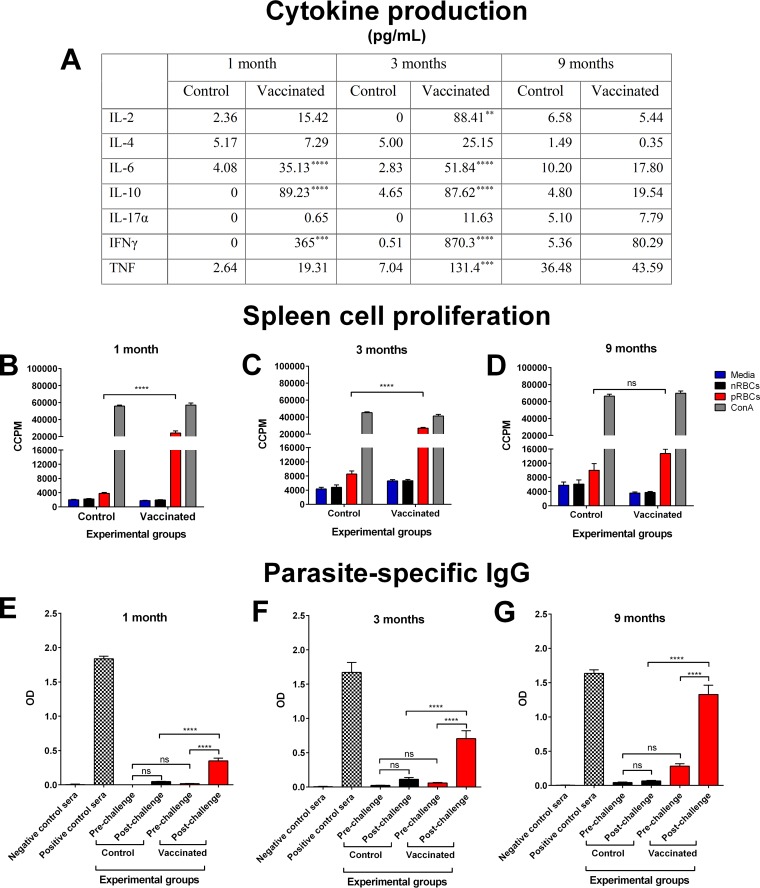
Vaccine-induced immune responses at 1, 3, and 9 months after completion of the vaccination regimen with chemically attenuated parasites. (A) Production of cytokines by splenocytes in response to homologous live pRBCs at 1, 3, and 9 months after completion of the three-dose vaccination regimen with 10^6^ chemically treated P. yoelii 17X pRBCs (vaccinated) or nRBCs (control). Culture supernatants from splenocyte proliferation assays were collected after 54 h and used in cytokine bead arrays to quantify the level of cytokines produced. The supernatant was pooled from triplicate wells for each mouse, and error bars represent the SEM for each group. Each group contained eight BALB/c mice. **, *P* = 0.0048; ***, *P* ≤ 0.0008; ****, *P* < 0.0001. (B to D) The proliferation of splenocytes from control and vaccinated BALB/c mice in response to fresh P. yoelii 17X pRBCs was assessed at 1 month (B), 3 months (C), or 9 months (D) after the final dose. The proliferation was estimated by the incorporation of [^3^H]thymidine and measured as corrected counts per minute (CCPM). Splenocytes from each mouse were tested in triplicate. Each group contained eight mice, and error bars represent the SEM for each group. ns, not significant. (E to G) Assessing parasite-specific memory B cell responses at 1 month (E), 3 months (F), and 9 months (G) after completion of the three-dose vaccination regimen. The production of IgG against crude P. yoelii 17X antigens was measured in the pre- and postchallenge sera of control and vaccinated BALB/c mice. Sera were titrated using 3-fold dilutions from a 1 in 50 dilution to a 1 in 109,350 dilution, with the 1 in 1,350 dilution shown as a representation of the data. Samples were tested in duplicate for each mouse. Sera from naive mice and infection-drug-cured mice were included as negative and positive controls, respectively. The results are expressed as the optical density (OD) at 450 nm. Each group contained ten mice, and error bars represent the SEM for each group. Data were analyzed using a one-way ANOVA, followed by Tukey's multiple-comparison tests.

Splenocytes were harvested from a cohort of vaccinated but unchallenged mice. Cytokines produced in response to parasite stimulation were examined. At 1 and 3 months after the final vaccination, there was significantly greater production of IL-6, IL-10, and IFN-γ by splenocytes from vaccinated mice than from control mice (*P* ≤ 0.0008) (Fig. 6A). In addition, at 3 months after the final vaccination, IL-2 and TNF production was also significantly higher (*P* ≤ 0.0048). At 9 months, there was no significant difference in any of the measured cytokines.

Spleen cells showed significantly higher parasite-specific proliferative responses than splenocytes from their respective control groups at 1 and 3 months after their final vaccination (*P* < 0.0001) (Fig. 6B and C). However, there was no significant difference in the proliferative response from vaccinated mice compared to control mice at 9 months (*P* = 0.2809) (Fig. 6D).

We next sought to determine whether B cell memory responses were induced by vaccination. Sera from vaccinated BALB/c mice were taken prior to challenge with P. yoelii 17X and on day 6 postchallenge for the 1-, 3-, and 9-month assay points. Sera from age-matched mice that received chemically treated nRBCs were also taken pre- and postchallenge at these time points. The stimulation of memory B cells is indicated by a rapid antibody response within 6 days of restimulation with antigen, whereas a primary immune response would take at least 9 days to produce parasite-specific IgG (36, 37).

Parasite-specific IgG was present in the prechallenge sera of vaccinated mice at a dilution of 1 in 20 at each time point compared to sera from control mice (data not shown). In order to assess a rapid antibody response indicative of stimulated memory B cells, a dilution of 1 in 1,350 was used. This was the lowest dilution across all time points at which there was no significant difference in the prechallenge parasite-specific IgG levels between control and vaccinated mice (1 month, *P* = 0.9357; 3 months, *P* = 0.9717; 9 months, *P* = 0.0837) (Fig. 6E to G). There was no significant difference in parasite-specific IgG in pre- and postchallenge sera from control mice at 1 month (*P* = 0.3816), 3 months (*P* = 0.6954), and 9 months (*P* = 0.9948). However, sera from vaccinated mice had significantly elevated parasite-specific IgG on day 6 postchallenge at the 1-, 3-, and 9-month time points compared to the levels prior to challenge at these time points (*P* < 0.0001), indicating a memory B cell response.

These data show that vaccination induced long-lasting immunological memory. An increase in parasite-specific IgG in vaccinated mice within 6 days after challenge demonstrates the induction of memory B cell responses that remained for at least 9 months after vaccination, whereas the ability of spleen cells to proliferate and produce cytokines in response to homologous parasite was sustained for at least 3 months after vaccination. This may indicate a more prominent role for B cells in the induction of long-lasting vaccine-induced protection.

### Stage-specific immunity.

**FIG 7:**
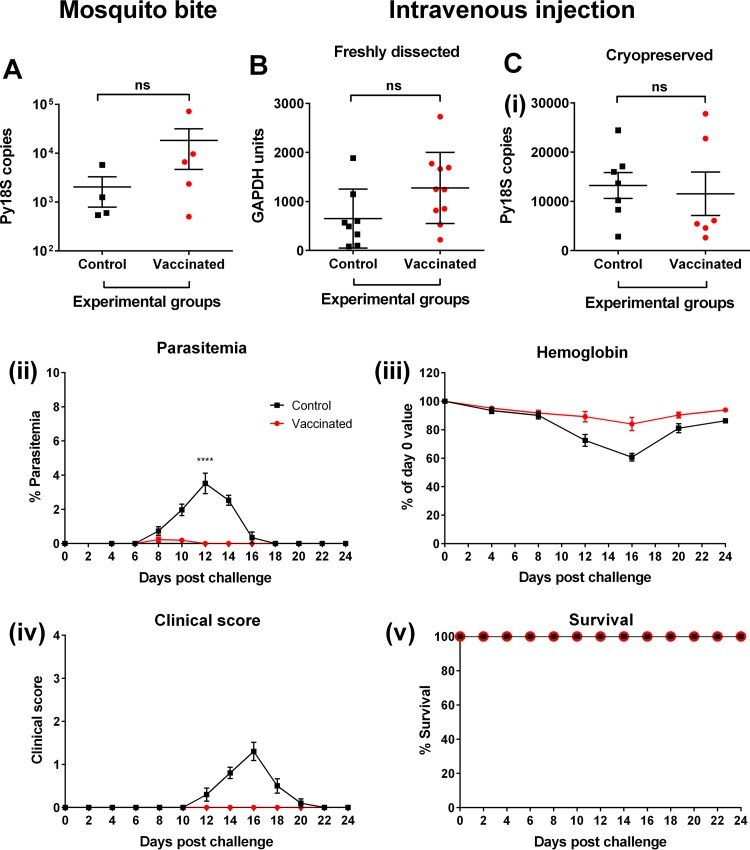
Assessing vaccine-induced stage-specific protection. (A to C) Parasite burden, as assessed by RT-qPCR in the livers of BALB/c mice that received four doses of 10^6^ chemically treated nRBCs (control; *n* = 4) or P. yoelii 17X pRBCs (vaccinated; *n* = 5) prior to challenge with ∼10 bites from P. yoelii 17X-infected mosquitoes (A), that received four doses of 10^6^ chemically treated nRBCs (control; *n* = 8) or P. yoelii 17X pRBCs (vaccinated; *n* = 10) prior to challenge with 2,000 freshly dissected P. yoelii 17X sporozoites (B), that received three doses of 10^6^ chemically treated nRBCs (control; *n* = 7) or P. yoelii 17X pRBCs (vaccinated; *n* = 6) prior to intravenous challenge with 4,000 cryopreserved P. yoelii 17X sporozoites (Ci). Error bars represent the SEM for each group. Depending on the variation in the level of the GAPDH housekeeping gene between samples, the parasite burden in the liver was expressed as Py18S copies or normalized and expressed as GAPDH units. (Cii to v) Assessment of parasitemia (Cii), hemoglobin levels (Ciii), clinical scores (Civ), and survival (Cv) in BALB/c mice that received three doses of 10^6^ chemically treated nRBCs or P. yoelii 17X pRBCs prior to intravenous challenge with 4,000 cryopreserved P. yoelii 17X sporozoites. Each group contained ten mice, and error bars represent the SEM for each group. The peak parasitemia was significantly lower for vaccinated mice than for control mice (****, *P* < 0.0001). Data were analyzed using an unpaired, two-tailed *t* test.

During natural infection, the blood stage of the life cycle is preceded by the liver stage. Here, CD8^+^ T cells are known to play a major role in protection (38, 39). Since activation of CD8^+^ T cells occurred postvaccination (Fig. 1A), we sought to determine whether vaccine-induced cross-stage protection against liver-stage parasites was induced by challenging BALB/c mice with sporozoites via mosquito bite or intravenous injection. After sporozoite challenge, livers from control and vaccinated mice were harvested and homogenized to assess parasite burden in the liver. RT-qPCRs were conducted for the Py18S parasite gene and the GAPDH housekeeping gene. After sporozoite challenge by mosquito bite, there was no significant difference in the parasite burden in the livers of vaccinated mice compared to control mice (*P* = 0.3277) (Fig. 7A). However, the sporozoite dose received by each mouse cannot be controlled due to the inherent variability of the number of sporozoites inoculated by mosquitoes (40–44). Therefore, protection was then assessed using intravenous administration of either freshly dissected or cryopreserved sporozoites; in both cases, there was no significant difference in parasite burden in the livers of vaccinated and control mice (*P* = 0.0686 and *P* = 0.7429, respectively) (Fig. 7B and Ci).

To assess the effect of vaccination on blood-stage infection after sporozoite challenge, a subset of mice that were challenged with the cryopreserved sporozoites were monitored through to the blood stage of the parasite life cycle (Fig. 7Ci). After challenge, peak parasitemia was reached on day 8 and day 12 for vaccinated (0.2311% ± 0.1548%) and control (3.52% ± 0.6042%) mice, respectively (Fig. 7Cii). Peak parasitemia was significantly lower in vaccinated mice than in control mice (*P* < 0.0001) (Fig. 7Cii). In addition, disease severity was much lower in vaccinated mice than in control mice, with vaccinated mice experiencing a less significant drop in hemoglobin levels (*P* = 0.003 versus *P* < 0.0001) (Fig. 7Ciii) and remaining healthy (with clinical scores of zero) (Fig. 7Civ). Mice from both groups survived (Fig. 7Cv).

## DISCUSSION

We investigated the protective efficacy of chemically attenuated P. yoelii blood-stage parasites. Vaccine-induced immunity appeared to be directed against blood-stage parasites (both homologous and heterologous strains), with no apparent effect against liver-stage parasites. Immunity was dependent on both cellular and humoral responses and was long-lived.

Investigations into the cellular immune responses induced by vaccination with chemically attenuated P. yoelii 17X showed activated circulating CD8^+^ T cells but only limited activation of circulating CD4^+^ T cells. However, further investigations into the role of CD4^+^ and CD8^+^ T cells in vaccine-induced protection demonstrated the crucial role of CD4^+^ T cells after blood-stage parasite challenge, with the depletion of CD4^+^ T cells resulting in a loss of protection. The limited activation of CD4^+^ T cells observed in the blood may indicate that these cells were located elsewhere, for example in the spleen. Although the role of CD4^+^ T cells in vaccine-induced protection was evident from previous depletion studies with a P. chabaudi chemically attenuated vaccine (20), the contribution of CD8^+^ T cells remained unclear. We show here for vaccination with P. yoelii-attenuated parasites that CD8^+^ T cells may contribute to pathogenesis. The data suggest that they require a higher parasite burden threshold to become pathogenic, because if parasitemia is patent, but controlled by CD4^+^ T cells, then the CD8^+^ T cells do not induce a deleterious response. The mechanism by which they mediate pathogenesis was not discerned, but a vigorous cytokine response is likely to play an important role (45). We previously demonstrated that vaccination of mice with attenuated P. chabaudi parasites led to a significant induction of IFN-γ-secreting CD8^+^ T cells (20), and it is known that this cytokine is implicated in pathogenesis (46, 47). However, to the best of our knowledge, a direct demonstration of CD8^+^ T cells promoting disease has not previously been described. Nevertheless, this scenario is at odds with other studies documenting a direct role for CD8^+^ T cells in controlling parasitemia (48, 49). In those studies, however, protection was demonstrated after the transfer of CD8^+^ T cells into normal mice in which CD4^+^ T cells were not depleted.

Cytokine analysis showed the production of both pro- and anti-inflammatory cytokines (IFN-γ, TNF, IL-2, IL-6, and IL-10) in vaccinated mice. Proinflammatory cytokines, such as IFN-γ and TNF, are involved in the resolution of malaria and are required for protection (50, 51). IL-10 is an anti-inflammatory and immunoregulatory cytokine produced by CD4^+^ T cells and is necessary to dampen the potentially pathogenic effects of proinflammatory cytokines, such as IFN-γ and TNF (52). The balance of these pro- and anti-inflammatory cytokines *in vivo* may limit disease severity and aid the quick resolution of blood-stage infection associated with vaccination. This may explain the exacerbation of immunopathology by CD8^+^ T cells in the absence of CD4^+^ T cells and CD4^+^ T cell-derived IL-10 (53).

The life cycle of the parasite has added complexity to the development of a malaria vaccine. However, shared antigenic targets (54, 55) between different life cycle stages may allow for the induction of cross-stage protection (17, 33, 56, 57), which would be valuable in the development of a malaria vaccine. Studies have shown that exposure to blood-stage parasites under chloroquine cover can induce cross-stage protection against liver-stage parasites after sporozoite challenge (17, 56). These studies are encouraging for the development of a whole-parasite blood-stage vaccine candidate capable of inducing cross-stage protection. However, our studies showed that vaccination with chemically attenuated blood-stage P. yoelii 17X parasites was unable to induce protection against mosquito bite or intravenous sporozoite challenge in terms of parasite burden in the liver, as assessed by qPCR. However, when infection was monitored through to the blood stage, vaccinated mice showed significantly lower blood-stage parasite burdens and disease severity than control mice.

During natural exposure to blood-stage Plasmodium infection, parasite-specific antibodies are acquired and predominate in protected individuals (2–4). Strong and long-lasting humoral immune responses, which are characterized by germinal center formation and long-lived plasma and memory B cells, have been shown to be dependent on CD4^+^ T cells with a follicular helper cell phenotype (58, 59). Unlike previous observations with three doses of chemically attenuated P. chabaudi blood-stage parasites (20), vaccination with three doses of chemically attenuated P. yoelii 17X blood-stage parasites did induce parasite-specific IgG. The difference in antibody response between the two vaccines may be due to the P. yoelii vaccine containing all the blood stages, whereas the P. chabaudi vaccine contained only ring-stage parasites. CD4^+^ T cells with a Tfh cell phenotype were present in higher numbers in the spleens of mice vaccinated with the P. yoelii vaccine than in spleens of control mice, suggesting the induction of T cell-dependent antibody responses (60). An investigation into the role of B cells in vaccine-induced protection was conducted using B cell-deficient mice. In the absence of B cells, vaccinated mice were unable to control parasite burden or disease severity, with outcomes similar to those for control mice after blood-stage infection. This study also showed the importance of vaccine-induced cellular immune responses in protection against mortality, since vaccinated B cell-deficient mice demonstrated prolonged survival after infection compared to control mice. However, mice in both groups eventually succumbed to infection.

An effective vaccine should aim to induce long-lasting protective immunity. Many malaria vaccine candidates have been unable to induce long-lasting protection during clinical evaluation in endemic settings (7, 8, 61). Vaccination with chemically attenuated P. yoelii 17X blood-stage parasites provided long-lasting protection against homologous blood-stage challenge that was sustained for at least 9 months. Memory B cells may have a role in this long-lasting vaccine-induced protection, since a significant elevation in parasite-specific IgG was detected during early infection in vaccinated mice. The kinetics of antibody production after challenge is indicative of a memory B cell response (37).

The development of a malaria vaccine also needs to reflect the multitude of Plasmodium strains and species that exist and are capable of causing disease in humans (1, 62, 63). A vaccine that is able to provide protection against multiple strains, and also multiple species of Plasmodium, would be advantageous. We have previously demonstrated the induction of cross-strain and species protection after vaccination with a chemically attenuated P. chabaudi vaccine (20). Here, we show that mice vaccinated with chemically attenuated P. yoelii 17X blood-stage parasites demonstrated robust protection against heterologous blood-stage infection with the lethal P. yoelii YM strain. Although the virulent P. yoelii YM strain was derived from an isolate of P. yoelii 17X (64), the courses of infection of the two strains are strikingly different. P. yoelii YM causes an extremely aggressive blood-stage infection in BALB/c mice, with death occurring within 10 days. Therefore, the complete protection against death and the resolution of P. yoelii YM infection in mice vaccinated with chemically attenuated P. yoelii 17X vaccine is encouraging for the development of this vaccine approach.

Rodent Plasmodium spp. are crucial in understanding aspects of human malaria and in the development of control measures against malaria. Our studies have demonstrated that chemically attenuated blood-stage P. yoelii 17X parasites can be used as a robust and effective vaccine against challenge with homologous and heterologous blood-stage P. yoelii parasites in mice. Long-lasting vaccine-induced protection and immunological responses were demonstrated against homologous parasites. These data are very encouraging for the development of an effective chemically attenuated blood-stage Plasmodium vaccine for use in humans.

## Supplementary Material

Supplemental material
