# Supplementary material for: Chemically Attenuated Blood-Stage Plasmodium yoelii Parasites Induce Long-Lived and Strain-Transcending Protection
Source: Infect Immun. 2016 Jul 21;84(8):2274–88. doi: 10.1128/IAI.00157-16 (PMC4962623; doi:10.1128/IAI.00157-16)
Supplement: Supplemental material [file supp_84_8_2274__index.html]

Chemically Attenuated Blood-Stage Plasmodium yoelii Parasites Induce Long-Lived and Strain-Transcending Protection — Supplemental material 

# Chemically Attenuated Blood-Stage Plasmodium yoelii Parasites Induce Long-Lived and Strain-Transcending Protection

## Supplemental material

- Supplemental file 1 -

  Fig. S1. Gating strategy to identify CD4+ T follicular helper cells. Fig. S2. Gating strategy to identify activated circulating CD4+ and CD8+ T cells following vaccination. Fig. S3. Gating strategy to identify effectiveness of CD4+ and CD8+ T cell depletion in spleens of vaccinated mice. Table S1. Clinical scoring system.

  PDF, 250K
