# Supplementary material for: Chemically Attenuated Blood-Stage Plasmodium yoelii Parasites Induce Long-Lived and Strain-Transcending Protection
Source: Infect Immun. 2016 Jul 21;84(8):2274–88. doi: 10.1128/IAI.00157-16 (PMC4962623; doi:10.1128/IAI.00157-16)
Supplement: Supplemental material [file IAI.00157-16_zii999091772so1.pdf]

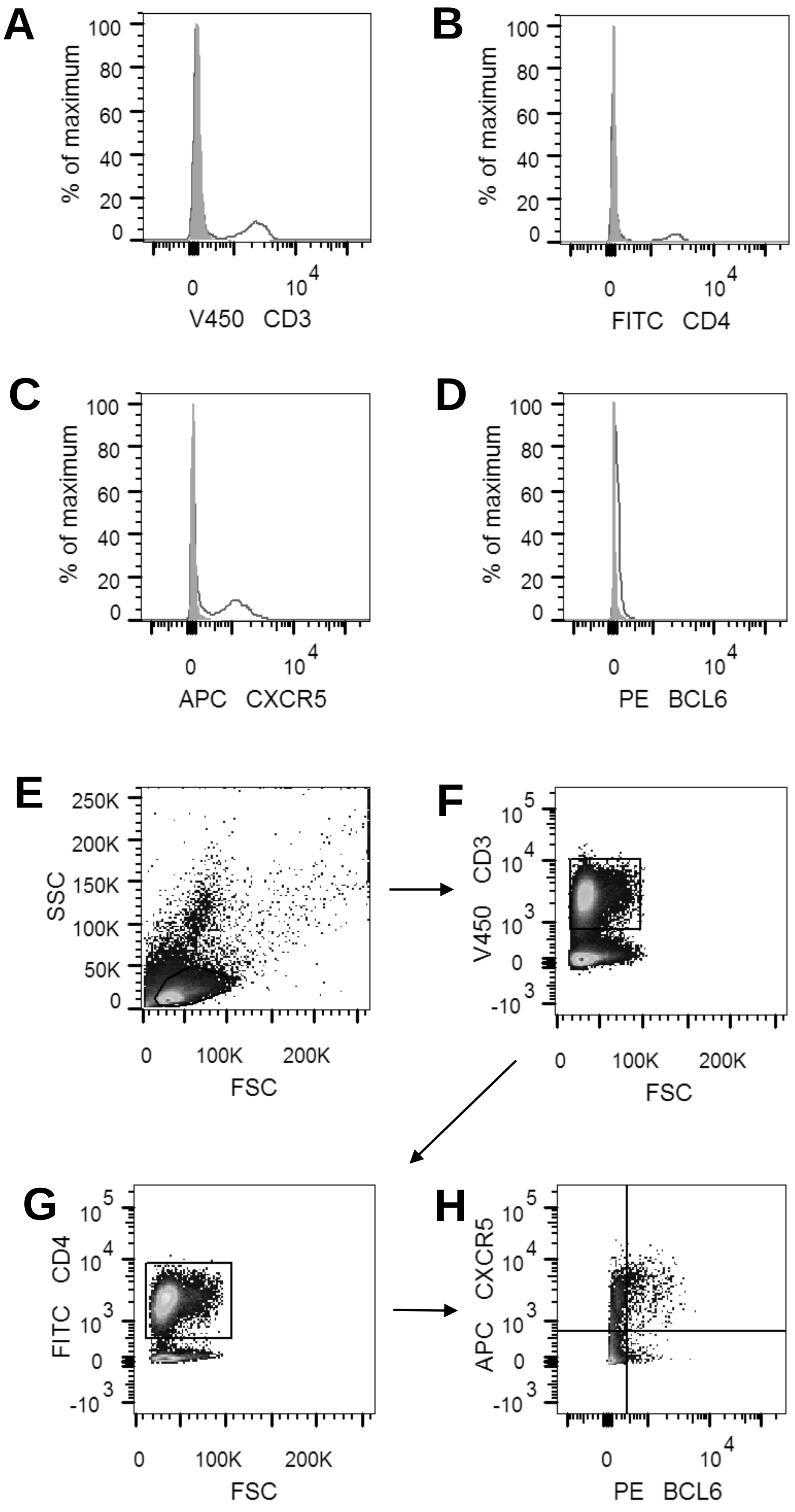
A
B
C
D
E
F
G
H
% of maximum
V450 CD3
FITC CD4
APC CXCR5
PE BCL6
SSC
FSC
FITC CD4
APC CXCR5

Figure S1. Gating strategy to identify $CD4^+$ T follicular helper (Tfh) cells. (**A-D**) Cells were identified as any fluorescence above that demonstrated by the isotype matched control antibodies. Gating used to identify (**E**) the lymphocyte population, (**F**) $CD3^+$ and (**G**) $CD4^+$ T cells. (**H**) Tfh cells were identified as $Bcl6^+CXCR5^+$.

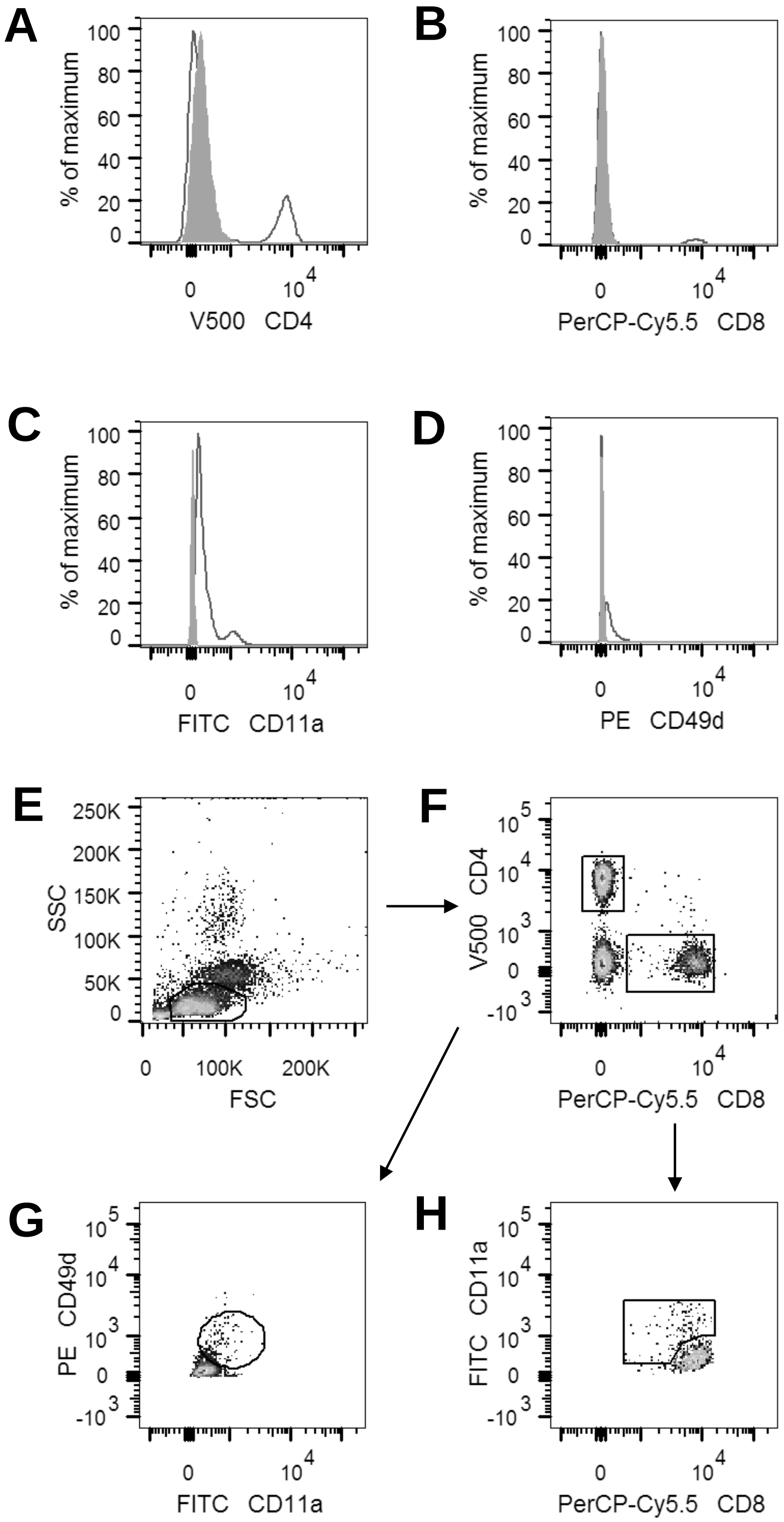
A
B
C
D
E
F
G
H
% of maximum
100
80
60
40
20
0
0
10^4
V500 CD4
PerCP-Cy5.5 CD8
FITC CD11a
PE CD49d
250K
200K
150K
100K
50K
SSC
FSC
100K
200K
10^5
10^3
-10^3
V500 CD4
PE CD49d
FITC CD11a

Figure S2. Gating strategy to identify activated circulating $CD4^+$ and $CD8^+$ T cells following vaccination. Activated $CD4^+$ and $CD8^+$ T cells were identified as any cells with fluorescence above that demonstrated by the isotype matched control antibodies (**A**-**D**). Gating was used to identify (**E**) the lymphocyte population, (**F**) $CD4^+$ and $CD8^+$ T cells, (**G**) activated $CD4^+$ T cells expressing $CD49d^{hi}CD11a^{hi}$ and (**H**) activated $CD8^+$ T cells expressing $CD8^{lo}CD11a^{hi}$.

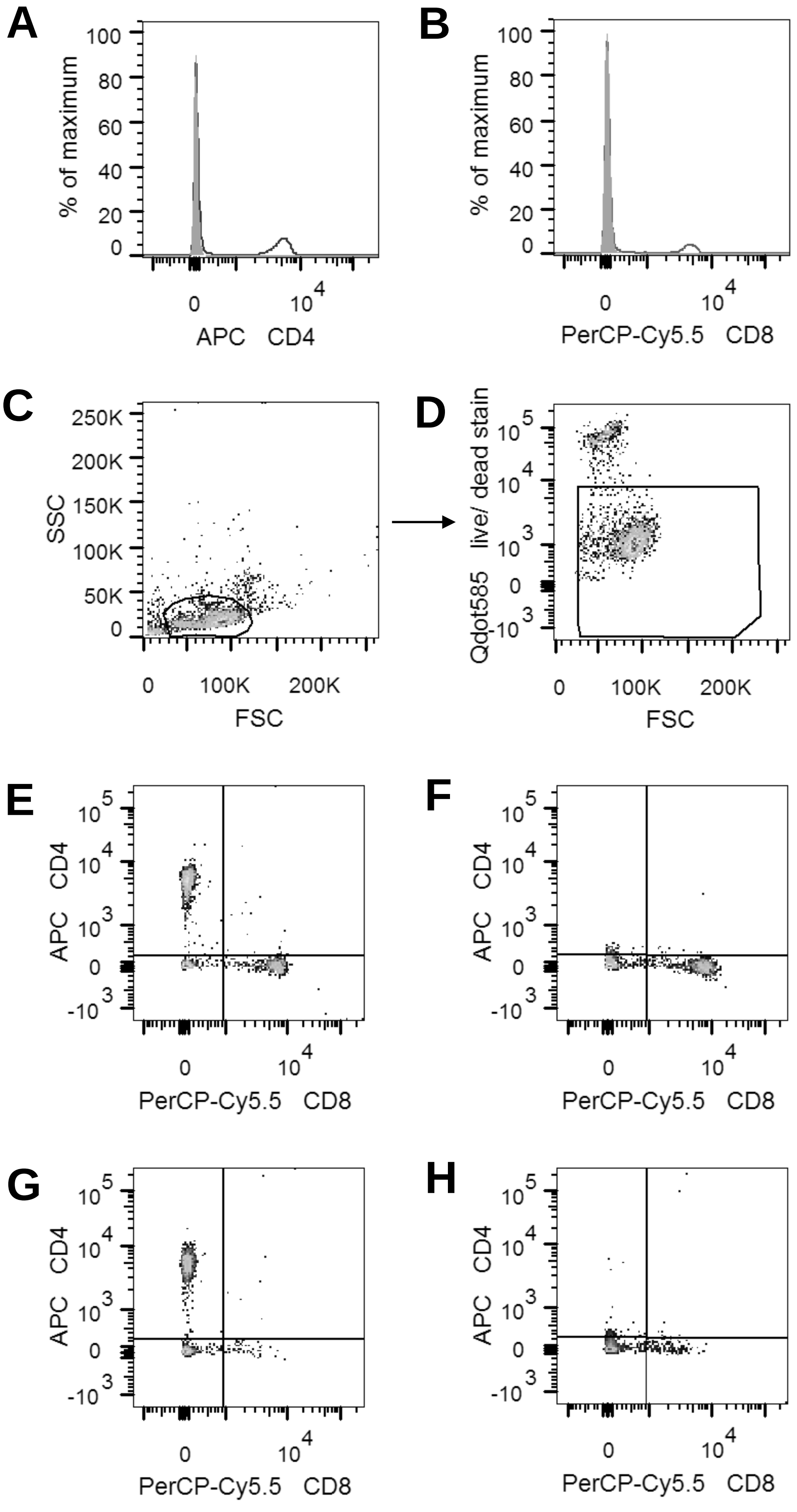
A
B
C
D
E
F
G
H
% of maximum
APC CD4
PerCP-Cy5.5 CD8
SSC
FSC
Qdot585 live/ dead stain

Figure S3. Gating strategy to identify effectiveness of $CD4^+$ and $CD8^+$ T cell depletion in spleens of vaccinated mice. (**A**) $CD4^+$ T cells and (**B**) $CD8^+$ T cells were identified as any cells with fluorescence above that demonstrated by the isotype matched control antibodies. Gating was used to identify (**C**) the lymphocyte population and (**D**) viable lymphocytes. Spleen cells from vaccinated mice that received 3 doses of 1 x 10^6 chemically attenuated *P. yoelii* 17X pRBCs before a course of intraperitoneal injections of (**E**) Rat Ig (control), (**F**) anti-CD4 antibodies (clone GK1.5), (**G**) anti-CD8 antibodies (clone 53.5.8) or (**H**) anti-CD4 and anti-CD8 antibodies.

Clinical Score	Checklist	Criteria
0		coat normal, glossy
		yellow urine
		good pallor: ankles, feet, toes and ears pink in color
		very active and running around
		gait and movement is smooth
		quick reaction to external stimuli
1	**If any of the following criteria**	
	A	change in urine color (slightly green or pale)
	B	slight pallor: pale feet or pale ankles
	C	slight hunched appearance
	D	slight ruffling of fur
2	**Score of 1 plus any of the following criteria**	
	E	increased pallor: includes pale white toes
	F	activity may be slightly decreased
	G	moderate ruffling of fur
3	**Score of 2 plus any of the following criteria**	
	H	pallor: very pale feet and/or ears
	I	urine orange
	J	severe ruffling of fur
	K	activity is moderately decreased but still moves upon stimulation
	L	reaction to external stimuli is slightly slowed - slow
4	**Score of 3 plus any of the following criteria**	
	M	extreme pallor: pale feet, ears, white toes
	N	urine bloody or dark green
	O	moderate-extreme hunching
	P	activity can be very reduced - stationary (just sits huddled in ball even with stimulation)
	Q	poor grooming may be present (feces stuck to fur or white matter near eyes)
	R	loss of muscle condition (feels like a bag of bones with loose skin when handling)
	S	splayed (spread) hind legs with or without head down (collapse) after handling
	T	failure to respond to stimuli

Table S1. Clinical scoring system. Disease severity was assessed using clinical scores on alternate days following challenge of vaccinated and control mice.
